# Association of low oleic acid intake with diabetic retinopathy in type 2 diabetic patients: a case–control study

**DOI:** 10.1186/s12986-016-0099-5

**Published:** 2016-06-04

**Authors:** Nuria Alcubierre, Eva M. Navarrete-Muñoz, Esther Rubinat, Mireia Falguera, Joan Valls, Alicia Traveset, Maria-Belen Vilanova, Josep Ramon Marsal, Marta Hernandez, Minerva Granado-Casas, Dolores Martinez-Gonzalez, Carmen Jurjo, Josep Franch-Nadal, Jesús Vioque, Didac Mauricio

**Affiliations:** Institut de Recerca Biomèdica de Lleida, University of Lleida, Lleida, 25198 Spain; Consortium for Biomedical Research in Epidemilogy and Public Health (CIBER en Epidemiología y Salud Pública CIBERESP), Madrid, 28029 Spain; Public Health Department, Miguel Hernandez University, Alicante, Spain; Unitat de Suport a la Recerca de Barcelona, Institut Universitari d’Investigació en Atenció. Primària Jordi Gol (IDIAP Jordi Gol), CIBER of Diabetes and Associated Metabolic Diseases (CIBERDEM), Barcelona, 08007 Spain; Primary Health Care Center Igualada Nord, Igualada, 08700 Spain; Biostatistics Unit, Institut de Recerca Biomèdica de Lleida, University of Lleida, Lleida, 25198 Spain; Department of Ophthalmology, University Hospital Arnau de Vilanova, Lleida, 25198 Spain; Unitat de Suport a la Recerca de Lleida, Institut Universitari d’Investigació en AtencióPrimària Jordi Gol (IDIAP Jordi Gol), Lleida, 25007 Spain; Unitat de Epidemiologia, Servicio de Cardiología, Hospital Universitari Vall d’Hebron, Barcelona, 08035 Spain; Department of Endocrinology and Nutrition, University Hospital Arnau de Vilanova, Lleida, 25198 Spain; Primary Health Care Center Raval Sud, Barcelona, 08001 Spain; Department of Endocrinology and Nutrition, University Hospital and Health Sciences Research Institute Germans Trias Pujol, CIBER of Diabetes and Associated Metabolic Diseases (CIBERDEM), Badalona, 08916 Spain

**Keywords:** Diabetic retinopathy, Oleic acid, Monounsaturated fatty acids, Fatty acids, Type 2 Diabetes mellitus

## Abstract

**Background:**

The objective of this study was to describe the intake of macronutrient, especially fatty acids, and explore their possible effect on diabetic retinopathy (DR) in patients with type 2 diabetes mellitus.

**Methods:**

In this case–control study, we included a total of 146 patients with DR and 148 without DR. The intake of macronutrient was evaluated using a validated food frequency questionnaire. We used logistic regression adjusted for sex, age, diabetes duration, energy intake, educational level, physical activity, waist circumference, systolic blood pressure, high-density lipoprotein cholesterol and diabetes treatment, to estimate odds ratio (ORs) of DR.

**Results:**

Patients with DR had significantly lower intake of fibre, monounsaturated fatty acids (MUFA), and palmitic and oleic acid. Inverse associations were observed between MUFA and oleic acid intake in DR. Subjects with intermediate and high MUFA intake were less likely to have DR than those with lower MUFA intake, with ORs of 0.46 (95 % CI: 0.22–0.93) and 0.42 (95 % CI: 0.18–0.97), respectively. Similarly, intermediate and high oleic acid intake were associated with reduced DR frequency compared with low oleic acid intake, with OR values of 0.48 (95 % CI: 0.23–0.97) and 0.37 (95 % CI: 0.16–0.85), respectively. These associations were stronger in patients with a longer diabetes duration.

**Conclusion:**

In type 2 diabetes mellitus, MUFA and oleic acid intake were inversely associated with DR.

## Background

Diabetic retinopathy (DR) is an ophthalmological complication of diabetes mellitus and is the main cause of non-recoverable vision loss in the working-age population [1]. In Spain, DR is still a relatively frequent diabetic microangiopathic complication [2].

The development of DR is determined by multiple contributing factors: duration and magnitude of hyperglycaemia, high blood pressure, dyslipidaemia, smoking, oxidative stress, inflammation, platelet dysfunction and several genetic factors [1, 3]. However, there is little evidence concerning the potential role of nutrients as protective factors against DR. Two intervention studies in humans showed a potential protective role of unsaturated fatty acids, especially linoleic acid, against the development and progression of DR in patients with type 2 diabetes mellitus (T2DM) [4, 5]. In a recently published post-hoc analysis from the PREDIMED intervention study, a Mediterranean diet supplemented with extra virgin olive oil showed a protective effect against retinopathy [6]. In a population-based cross-sectional study in patients with T2DM in India, low fibre intake was identified as a risk factor for the development of DR [7]. Tanaka et al. demonstrated that increased fruit consumption is associated with a lower incidence of DR in a cohort of Japanese patients with T2DM who followed a low-fat, energy-restricted diet [8]. In a small cross-sectional study, diabetic patients without retinopathy had higher fibre and carbohydrate intake and a lower proportion of dietary energy from protein than their counterparts with retinopathy [9]. However, these results are far from consistent.

In this study, we describe the intake of macronutrients, especially fatty acids, and explore their association with diabetic retinopathy (DR).

## Methods

### Study design and subjects

In this case–control study, we included a total of 146 type 2 diabetic patients with DR and 148 type 2 diabetic patients without DR. All of the participants were aged 40 to 75 years. The primary recruitment of the cases took place within the previously published diabetic retinopathy screening and treatment program of the Department of Ophthalmology [10]. Participants in that former study were offered dietary questionnaires to participate in this study, and only 5 declined (response rate: 98.3 %). Retinopathy status was assessed and classified by an experienced ophthalmologist following an international clinical classification system [11]. The presence of the following advanced diabetes complications was an exclusion criterion: renal disease (macroalbuminuria or renal insufficiency) and cardiovascular disease. These exclusion criteria were defined in the initial design of the study as they are known to affect quality of life which was one of the main outcomes of the original study [10]. The recruitment process took place between March 2010 and January 2013. The local ethics committee approved the study, and all study subjects signed a written informed consent form.

### Clinical and socio-demographic variables

A detailed description of the methodology of assessment of the clinical variables was recently published [10]. The following socio-demographic and clinical characteristics were collected: age, sex, diabetes duration, self-reported ethnic group, smoking habits, physical activity, level of education, blood pressure, lipid profile, antidiabetic treatments, antihypertensive and hypolipidaemic treatments, and glycated haemoglobin (HbA1c).

### Dietary data

Food and nutrient intake was assessed using a 101-food item semiquantitative food frequency questionnaire (FFQ) (available at: http://bibliodieta.umh.es/cfa-101-inma). The FFQ was an adapted version of the questionnaire by Willett et al. [12] and validated for Spanish populations [13]. The mean correlation coefficients between the nutrient intakes estimated using prospectively collected diet records and those estimated with the FFQ were 0.47 for validity and 0.40 for reproducibility [14]. The questionnaires were released to and controlled by one of the researchers (N.A.) on an individual basis. Participants were asked to report how often, on average, they had consumed each food item over the past year. Serving sizes were specified for each food item in the food frequency questionnaire (FFQ). The questionnaire offered nine options for frequency of consumption for each food, ranging from “never or less than once a month” to “6 or more times/day”. Nutrient values for each food in the questionnaire were mainly obtained from food composition tables of the US Department of Agriculture and supplemented with Spanish sources [15, 16].

### Sample size

The inclusion of a minimum of 146 individuals in each group (DR and no DR patients) was sufficient to achieve an 80 % statistical power (β = 0.2) with a 5 % significance level (α = 0.05) to detect a difference of at least 40 g in the carbohydrate intake, 81 mg in cholesterol intake, and 3.2 g in fibre intake (assuming standard deviations of 104.5, 215.0 and 8.3, respectively). The data for standard deviations were based on the results published in a previous study conducted in Spain [17].

### Statistical analysis

Means (and standard deviations) and absolute and relative frequencies (in percentages) were computed for quantitative and qualitative variables, respectively. The differences in the clinical and sociodemographic characteristics between the DR and no DR patients were assessed using a Chi-squared test or Fisher’s exact test for comparison of qualitative variables and Student’s t test for comparison of quantitative variables. Logistic regression analyses were fitted to assess the association between the macronutrient intake, especially fatty acids, and DR adjusted for sex, age, diabetes duration and energy intake. In a subsequent model we also adjusted for all significant covariates in the univariate analysis (*p* value < 0.20), such as educational level, physical activity, waist circumference, systolic blood pressure, high-density lipoprotein cholesterol (HDL-cholesterol) and diabetes treatment. Tests for linear trends in the tertiles of nutrient intake were evaluated introducing the variable (1, 2, 3) as a continuous term in the logistic regression models. To explore the effect of the duration of diabetes (categorization based on a median diabetes duration ≤ 8 years vs > 8 years) on the association between macronutrients and DR, the models were stratified by duration of diabetes.

## Results

Table 1 shows the clinical and socio-demographic characteristics of the study participants according to DR status. Patients with DR were older and had a lower education level. Patients with DR also had a longer diabetes duration, were more frequently treated with insulin, and had higher HbA1c concentrations than patients without DR.

**Table 1:** Clinical characteristics of patients with diabetic retinopathy (DR) and patients with no retinopathy (no DR)

Characteristics	No DR (*n* = 148)	DR (*n* = 146)	*p* value^*^
Age (years), mean (SD)	57.9 (10.3)	60.5 (8.8)	0.021
Men, % (*n*)	52.0 (77)	50.0 (73)	0.816
Non Caucasian, % (*n*)	3.4 (5)	4.1 (6)	0.769
Educational level, % (*n*)			0.001
≤ Primary	60.8 (90)	77.4 (113)	
Secondary	27.0 (40)	20.5 (30)	
University	12.2 (18)	2.1 (3)	
Tobacco use, % (*n*)			0.629
Never smoker	45.3 (67)	50.7 (74)	
Former smoker	33.8 (50)	29.5 (43)	
Current smoker	20.9 (31)	19.9 (29)	
Physical activity,% (*n*)			0.045
Active	66.2 (98)	55.9 (81)	
Sedentary	33.8 (50)	44.1 (64)	
HbA1c (%), mean (SD)	7.3 (1.2)	8.3 (1.4)	< 0.001
Diabetes duration (years), mean (SD)	7.1 (5.5)	14.1 (9.9)	< 0.001
Body mass index, Kg/m^2^, mean (SD)	31.3 (5.1)	32.0 (5.5)	0.273
Waist circumference (cm), mean (SD)	104.2 (11.9)	107.8 (11.9)	0.010
Systolic blood pressure (mmHg), mean (SD)	134.3 (15.5)	145.0 (19.9)	< 0.001
Diastolic blood pressure (mmHg), mean (SD)	76.5 (10.5)	77.1 (11.1)	0.628
Total cholesterol (mg/dL), mean (SD)	185.7 (36.6)	184.8 (36.1)	0.822
HDL-cholesterol (mg/dL), mean (SD)	48.5 (10.7)	52.1 (15)	0.021
LDL-cholesterol (mg/dL), mean (SD)	111 (30.7)	106.9 (30.1)	0.182
Triglycerides (mg/dL)	138.6 (81.8)	140.5 (120)	0.879
Diabetes treatment, % (*n*)			< 0.001
Oral agents	64.2 (95)	43.8 (64)	
Insulin + oral agents	8.8 (13)	41.8 (61)	
Insulin	2.7 (4)	12.3 (18)	
Diet only	24.3 (36)	2.1 (3)	

^*^ *p*-value from Chi-squared or Fisher’s exact tests for comparison of qualitative variables and Student’s t tests for comparison of quantitative variables. *SD* standard deviation, *HbA1c* glycated haemoglobin, *HDL*-*cholesterol* high-density lipoprotein cholesterol, *LDL*-*cholesterol* low-density lipoprotein cholesterol

The mean daily nutrient intakes were very similar in patients with DR and patients without DR (Table 2). However, the patients with DR showed a lower intake of fibre, monounsaturated fatty acids, and palmitic and oleic acid.

**Table 2:** Mean daily nutrient intake of patients with diabetic retinopathy (DR) and those without retinopathy (no DR)

Nutrient intake	No DR (*n* = 148)	DR (*n* = 146)	*p*-value^*^
Energy intake (kcal)	2198 (581)	2088 (581)	0.105
Protein (g)	108.3 (30.1)	107.1 (37.4)	0.776
Carbohydrate (g)	232 (76)	222.1 (79.6)	0.275
Fibre (g)	28.3 (8)	26.3 (7.4)	0.032
Total fat (g)	92 (27)	85.7 (28.8)	0.054
Saturated fatty acids (g)	25.2 (9)	23.1 (10)	0.052
Monounsaturated fatty acids (g)	43.6 (12.8)	39.9 (14.2)	0.018
Polyunsaturated fatty acids (g)	16.1 (7.0)	16 (7.7)	0.842
Omega 3 (g)	1.77 (0.71)	1.78 (1.05)	0.921
Omega 6 (g)	14.2 (6.6)	14 (7.2)	0.817
Trans fat (g)	1.13 (0.94)	1.05 (0.97)	0.503
Palmitic acid (g)	15.1 (4.7)	13.9 (5.2)	0.043
Stearic acid (g)	5.92 (2.16)	5.49 (2.83)	0.145
Oleic acid (g)	41.2 (12.2)	37.5 (13.5)	0.016
Linoleic acid (g)	14.1 (6.6)	13.9 (7.2)	0.813

^*^ *p*-value for Student’s *t* test. All values are means (SD). SD: standard deviation

The adjusted logistic regression analysis revealed that there was an inverse linear association between monounsaturated fatty acid intake tertiles and DR (*p* = 0.034) and between oleic acid intake tertiles and DR (*p* = 0.017). The fully adjusted ORs (95%CIs) for categories of intermediate and high monounsatured fatty acid consumption compared with the low consumption were 0.46 (0.22–0.93) and 0.42 (0.18–0.97), respectively. In addition, adjusted ORs (95 % CIs) for categories of intermediate and high oleic acid consumption compared with the low consumption were 0.48 (0.23–0.97) and 0.37 (0.16–0.85), respectively. Assessing monounsaturated fatty acid and oleic acid consumption as continuous variables, there was evidence of an inverse association, but these associations were of borderline significance (Table 3).

**Table 3:** Association between macronutrient intake and diabetic retinopathy (DR) in case–control study

Nutrient intake		No DR/DR	OR^a^ (95 % CI)	*p*-trend^b^	OR^c^ (95 % CI)	*p*-trend^b^
Energy intake (kcal)	Per 1000 cals	148/146	0.81 (0.52; 1.26)		0.76 (0.48; 1.23)	
	≤ 1849	46/52	1.00	*0.364*	1.00	*0.377*
	1850 – 2272	47/51	0.77 (0.41; 1.45)		0.80 (0.40; 1.59)	
	≥ 2273	55/43	0.75 (0.40; 1.40)		0.73 (0.37; 1.46)	
Protein (g)	Per 10 g	148/146	1.03 (0.92; 1.14)		1.03 (0.91;1.17)	
	≤ 92.9	48/49	1.00	*0.865*	1.00	*0.645*
	93.0–117.6	51/48	0.98 (0.51; 1.90)		1.15 (0.56; 2.37)	
	≥ 117.7	49/49	1.09 (0.46; 2.56)		1.24 (0.49; 3.16)	
Carbohydrate (g)	Per 100 g	148/146	1.49 (0.80; 2.77)		1.58 (0.80; 3.12)	
	≤ 189.6	45/53	1.00	*0.859*	1.00	*0.729*
	189.7–234.0	47/51	0.95 (0.49; 1.82)		1.09 (0.53; 2.23)	
	≥ 234.1	56/42	0.93 (0.39; 2.21)		1.18 (0.45; 3.09)	
Fibre (g)	Per 10 g	148/146	0.74 (0.47;1.15)		0.83 (0.50; 1.36)	
	≤ 23.4	41/56	1.00	*0.301*	1.00	*0.421*
	23.5–29.2	51/48	0.67 (0.36; 1.27)		0.55 (0.28; 1.11)	
	≥ 29.3	56/42	0.69 (0.33; 1.46)		0.76 (0.33; 1.76)	
Total fat (g)	Per 10 g	148/146	0.83 (0.69;1.01)		0.86 (0.70; 1.06)	
	≤ 76.3	43/54	1.00	*0.194*	1.00	*0.275*
	76.4 – 96.2	52/46	0.53 (0.27; 1.06)		0.59 (0.27; 1.25)	
	≥ 96.3	53/46	0.58 (0.24; 1.39)		0.60 (0.23; 1.55)	
Saturated fatty acids (g)	Per 10 g	148/146	0.56 (0.33; 0.95)		0.64 (0.36; 1.14)	
	≤ 19.2	45/53	1.00	*0.052*		*0.133*
	19.3 – 26.6	45/52	0.81 (0.41; 1.61)		0.63 (0.30; 1.33)	
	≥ 26.7	58/41	0.42 (0.18; 1.00)		0.49 (0.19; 1.26)	
Mono-unsaturated fatty acids (g)	Per 10 g	148/146	0.76 (0.58; 1.01)		0.74 (0.55;1.00)	
	≤ 36.0	39/59	1.00	*0.050*	1.00	*0.034*
	36.1 – 46.2	53/45	0.53 (0.28; 1.01)		0.46 (0.22; 0.93)	
	≥ 46.3	56/42	0.49 (0.23; 1.03)		0.42 (0.18; 0.97)	
Poly-unsaturated fatty acids (g)	Per 10 g	148/146	1.12 (0.72;1.74)		1.27 (0.80;2.02)	
	≤ 12.3	45/53	1.00	*0.804*	1.00	*0.900*
	12.4 – 16.6	52/46	0.74 (0.38; 1.45)		0.84 (0.41; 1.75)	
	≥ 17.7	51/47	0.91 (0.40; 2.07)		1.07 (0.44; 2.64)	
Omega 3 (g)	Per 1 g	148/146	1.11 (0.80; 1.55)		0.99 (0.69; 1.41)	
	≤1.43	50/48	1.00	*0.909*	1.00	*0.927*
	1.44 – 1.91	47/51	1.14 (0.61; 2.15)		1.25 (0.62; 2.52)	
	≥ 1.92	51/47	0.95 (0.45; 2.00)		0.95 (0.41; 2.16)	
Omega 6 (g)	Per 10 g	148/146	1.09 (0.70; 1.71)		1.27 (0.79;2.05)	
	≤10.3	43/55	1.00	*0.634*	1.00	*0.851*
	10.4 – 14.8	56/42	0.61 (0.31; 1.19)		0.77 (0.37; 1.60)	
	≥ 14.9	49/49	0.83 (0.37; 1.87)		1.10 (0.45; 2.67)	
Trans fat (g)	Per 1 g	148/146	1.03 (0.73; 1.45)		1.12 (0.76; 1.65)	
	≤ 0.61	41/56	1.00	*0.325*	1.00	*0.810*
	0.62 – 1.09	54/45	0.64 (0.34; 1.22)		0.64 (0.32; 1.31)	
	≥1.10	53/45	0.70 (0.38; 1.46)		0.93 (0.41; 2.07)	
Palmitic acid (g)	Per 10 g	148/146	0.27 (0.09; 0.80)		0.32 (0.10; 1.06)	
	≤12.1	44/53	1.00	*0.029*	1.00	*0.077*
	12.2 – 15.8	45/54	0.82 (0.42; 1.60)		0.67 (0.32; 1.41)	
	≥15.9	59/39	0.35 (0.14; 0.87)		0.41 (0.15; 1.01)	
Stearic acid (g)	Per 1 g	148/146	0.92 (0.76; 1.12)		0.98 (0.81; 1.20)	
	≤4.52	41/56	1.00	*0.050*	1.00	*0.131*
	4.53 – 6.26	52/47	0.55 (0.28; 1.09)		0.43(0.20; 0.92)	
	≥6.27	55/43	0.43 (0.18; 1.00)		0.49 (0.19; 1.25)	
Oleic acid (g)	Per 10 g	148/146	0.76 (0.57; 1.00)		0.74 (0.54; 1.01)	
	≤32.2	38/59	1.00	*0.019*	1.00	*0.017*
	32.3 – 43.6	52/47	0.56 (0.29; 1.06)		0.48 (0.23; 0.97)	
	≥43.6	58/40	0.41 (0.20; 0.88)		0.37 (0.16; 0.85)	
Linoleic acid (g)	Per 10 g	148/146	1.09 (0.70; 1.71)		1.27 (0.79; 2.05)	
	≤10.3	43/55	1.00	*0.512*		*0.909*
	10.4 – 14.7	55/43	0.61 (0.31; 1.19)		0.77 (0.37; 1.61)	
	≥14.8	50/48	0.77 (0.35; 1.72)		0.95 (0.39; 2.31)	

^a^Adjusted for sex, age, diabetes duration and energy intake. ^b^ *p* value for linear trends examined using likelihood ratio tests. ^c^Adjusted for sex, age, diabetes duration, energy intake, educational level, physical activity, waist circumference, systolic blood pressure, HDL-cholesterol and diabetes treatment. *No DR* Diabetic no retinopathy, *DR* diabetic retinopathy, *OR* Odds ratio

In table 4, the results of the analyses of the association of nutrient intake with DR stratified by the diabetes duration (≤ 8 years vs > 8 years) are shown. The OR values did not materially change in the associations, but the intakes of monounsaturated fatty acids and oleic acid were inversely associated with DR in the participants with a duration of diabetes > 8 years.

**Table 4:** Odds ratios (OR) and corresponding 95 % confidence interval (CI) of diabetic retinopathy (DR) by nutrient intake according to duration of diabetes

		All cases (148/146)	Duration of diabetes≤ 8 years (102/53)	Duration of diabetes> 8 years (46/93)
Nutrient intake		OR^a^ (95 % CI)	OR^a^ (95 % CI)	OR^a^ (95 % CI)
Energy intake (kcal)	Per 1000 cal	0.76 (0.48; 1.23)	0.76 (0.41; 1.39)	0.85 (0.39; 1.86)
Protein (g)	Per 10 g	1.03 (0.91; 1.17)	1.05 (0.90; 1.21)	1.07 (0.82; 1.39)
Carbohydrate (g)	Per 100 g	1.58 (0.80; 3.12)	0.86 (0.34; 2.20)	3.05 (0.96; 9.72)
Fibre (g)	Per 10 g	0.83 (0.50;1.36)	0.42 (0.19; 0.93)	1.44 (0.69; 3.03)
Total fat (g)	Per 10 g	0.86 (0.70; 1.06)	1.08 (0.80; 1.47)	0.70 (0.51; 0.97)
Saturated fatty acids (g)	Per 10 g	0.64 (0.36; 1.14)	0.83 (0.40; 1.72)	0.43 (0.18; 1.05)
Mono-unsaturated fatty acids (g)	Per 10 g	0.74 (0.55; 1.00)	1.17 (0.76; 1.80)	0.49 (0.30; 0.81)
Poly-unsaturated fatty acids (g)	Per 10 g	1.27 (0.80; 2.02)	1.07 (0.54; 2.11)	1.42 (0.69; 2.91)
Omega 3 (g)	Per 1 g	0.99 (0.69; 1.41)	1.23 (0.71; 2.14)	0.59 (0.25; 1.39)
Omega 6 (g)	Per 10 g	1.27 (0.79; 2.05)	1.01 (0.50; 2.04)	1.50 (0.71; 3.17)
Trans fat (g)	Per 1 g	1.12 (0.76; 1.65)	1.24 (0.67; 2.30)	0.95 (0.54; 1.67)
Palmitic acid (g)	Per 10 g	0.33 (0.10;1.06)	1.03 (0.23; 4.71)	0.10 (0.01; 0.62)
Stearic acid (g)	Per 1 g	0.98 (0.81;1.20)	1.05 (0.84; 1.32)	0.80 (0.55; 1.17)
Oleic acid (g)	Per 10 g	0.74 (0.54;1.01)	1.16 (0.74; 1.80)	0.49 (0.29; 0.81)
Linoleic acid (g)	Per 10 g	1.27 (0.79;2.05)	1.01 (0.50; 2.03)	1.50 (0.71; 3.18)

^a^Adjusted for sex, age, diabetes duration, energy intake, educational level, physical activity, waist circumference, systolic blood pressure, HDL-cholesterol and diabetes treatment. *CI* interval confidence, *OR* Odds ratio

## Discussion

The findings of the present study indicate that in type 2 diabetic patients from our region, the presence of diabetic retinopathy is related to a lower intake of oleic acid. Low oleic acid intake showed a clear relationship with diabetes duration, revealing that the association between the low intake of this nutrient in patients with retinopathy increased as the disease duration lengthened. However, in a study with a cross-sectional design it is not possible to differentiate the relative contributions of disease duration and specific dietary intakes. Additionally, the effect of long-term hyperglycemia to the development of DR can only be explored in a long-term prospective study. Oleic acid represents more than 90 % of the monounsaturated fatty acid intake of these patients, with the consumption of olive oil being the main source in Spain. Although the patients with retinopathy also showed a lower saturated fat intake, this association did not reach statistical significance.

A potential role of unsaturated fatty acids was initially described in two intervention studies carried out in the 1980s [4, 5]. The first study showed a preventive effect of a linoleic acid – rich diet on the development of microangiopathy and the progression of diabetic retinopathy in T2DM [4]. The second intervention study confirmed the protective role of a high polyunsaturated fatty acid diet compared with a low-carbohydrate diet in the development of retinopathy in T2DM patients [5]. A recent post hoc analysis of a cohort of patients with type 2 diabetes in the PREDIMED trial described a potential protective effect between MedDiet supplemented with extra virgin olive oil and prevention of diabetic retinopathy [6]. Our results also illustrate an association between the presence of DR and intake of monounsaturated fatty acids and oleic acid in patients with T2DM, without other late diabetic complications, in the same geographical area. A previous prospective study in Spain showed the potential protective role of polyunsaturated and monounsaturated fatty acids in the development of diabetic complications in a small sample of patients with type 1 and type 2 diabetes [18] Olive oil is one of the main components of the Mediterranean diet, a diet that has demonstrated a significant effect in the prevention of cardiovascular disease [19]. Current and previous results point to the protective role that poly- and monounsaturated fatty acids may have, not only on macrovascular but also on microvascular beds of diabetic subjects. Additionally, although oleic acid is the main component of olive oil, many other substances in this fat source, especially anti-oxidants and anti-inflammatory polyphenols, could play a neuroprotective role in the retina [20–22]. A higher consumption of extra virgin olive oil, with a high content in oleic acid and polyphenols, could have exerted anti-inflammatory effects with a beneficial impact on the onset and progression of retinal damage.

Concerning other dietary fat components, we did not find an association with omega-3 fatty acid intake or polyunsaturated fatty acids. Although these fatty acids have been shown to have anti-inflammatory properties, potentially involved in the pathogenesis of DR, we could not find an association. The results showed a lower palmitic acid intake also in patients with DR. This seems to be related, at least in part, to a lower intake of saturated fat in patients with DR. However, after adjustment for potential confounding variables, this association disappeared (Table 3). Nevertheless, we can not rule out a potential association of other components of dietary fat in the development of retinopathy, especially in populations with different patterns of fat intake.

Previous studies have described associations of fruit consumption, higher fibre and carbohydrate intake, and lower protein intake with a protective effect on retinopathy [7–9]. However, we were not able to show any difference in protein, fibre or carbohydrate intake between the patients with and without retinopathy after adjusting for confounding factors. Except for the three intervention studies mentioned above, all other studies in this area, including ours, have been cross-sectional. Therefore, prospective studies are needed, as are intervention trials using olive oil nutritional supplementation to target populations with diabetic retinopathy and other eye diseases.

The main limitation of our study is its cross-sectional design, which prevents the adoption of any conclusions about the causality of any nutritional factors in retinopathy. We also acknowledge that patients with DR had distinct clinical characteristics. Some of these clinical features are intrinsically associated with diabetic retinopathy, e.g., hypertension and longer diabetes duration, which is in turn associated with poorer glycaemic control and more complex hypoglycaemic treatment regimens. Additionally, we were unable to detect other habits or external factors that may have been associated with the lower consumption of specific nutrients. However, the clinical characteristics of the studied patients are similar to those recently described in our region [2]. Additionally, it should be noted that this study was performed in patients from just one province in Spain; thus, the current results may not be generalized to other populations with a different pattern of monounsaturated fatty acid and/or oleic acid intake. Finally, the dietary intake was evaluated through a FFQ that evaluates foot intake only over the previous one-year period; however, we have shown that this FFQ is representative of previous longer time period food intake (up to 5 years) [13].

## Conclusions

In conclusion, a low intake of monounsaturated fatty acids and oleic acid is associated with the presence of retinopathy in type 2 diabetic patients from the Mediterranean region. These and previous findings highlight the potential role of nutrients in the prevention of the development and/or progression of diabetic retinopathy. Further research is needed in this area, especially research based on prospective studies.

## Abbreviations

CI, Confidence interval; DR, Diabetic retinopathy; FFQ, Food frequency questionnaire; FFQ, Food frequency questionnaire; HbA1C, Glycated haemoglobin; HDL-cholesterol, High-density lipoprotein cholesterol; MUFA, Monounsaturated fatty acids; OR, Odds ratio; SD, Standard deviation; T2DM, Type 2 diabetes mellitus
